# High Prevalence of Gastroesophageal Reflux Disease in Parkinson's Disease: A Questionnaire-Based Study

**DOI:** 10.1155/2013/742128

**Published:** 2013-02-11

**Authors:** Tetsuya Maeda, Ken Nagata, Yuichi Satoh, Takashi Yamazaki, Daiki Takano

**Affiliations:** Department of Neurology, Research Institute for Brain and Blood Vessels, 6-10 Senshu-Kubota-Machi, Akita 010-0874, Japan

## Abstract

The aim of this study is to investigate the frequency and clinical features of gastroesophageal reflex disease (GERD) in Parkinson's disease (PD). Consecutively recruited PD patients and controls were questioned about heartburn and GERD with a questionnaire. In PD patients, disease duration and severity, quality of life, and nonmotor symptoms were also examined and then the clinical features of GERD were analyzed. A total of 102 patients and 49 controls were enrolled and 21 patients and 4 controls had heartburn, significantly frequent in PD. The prevalence rate of GERD was 26.5% in PD and the odds ratio was 4.05. Heartburn, bent forward flexion, and wearing-off phenomenon were frequent, and scores of UPDRS, total and part II, PD questionnaire-39, and nonmotor symptom scale were significantly higher in PD patients with GERD than without GERD. Multiple logistic regression analysis revealed statistical significance in UPDRS part II and nonmotor symptom scale. This study suggests that GERD is prevalent in PD. Deterioration of daily living activities and other nonmotor symptoms can imply the presence of GERD. Because clinical symptoms of GERD are usually treatable, the management can improve the patient's quality of life. Increased attention should be given to detect GERD in PD.

## 1. Introduction

Gastroesophageal reflux symptoms characterized by heartburn and regurgitation are generally recognized as clinical symptoms of gastroesophageal reflex disease (GERD). GERD can also show dyspeptic manifestations other than reflux symptoms. In clinical practice, disappearance of these symptoms following treatment with proton pump inhibitors (PPIs) allows general physicians to reasonably conclude that the patient had acid-related dyspepsia [1]. Dyspepsia is usually defined as upper abdominal pain or retrosternal pain, discomfort, heartburn, nausea, vomiting, or other symptoms considered to arise from the upper alimentary tract. When these symptoms cause deterioration of patients' daily life quality, PPIs are generally used for treatment because they are more effective than histamine H2 receptor antagonists for reflux-like (heartburn) or ulcer-like (episodic epigastric pain) dyspepsia.

Gastrointestinal dysfunction is one of the most common nonmotor features of Parkinson's disease (PD), from the original description by James Parkinson. Variable abnormalities from the mouth through the rectum are already known [2]. Dysphagia is relatively common and observed in 29%–80% of PD patients [2, 3], which can be induced by dyscoordination of various organs such as the mouth, pharynx, and esophagus. In addition to abnormalities of esophageal peristalsis, dysfunction in the lower esophageal sphincter can also produce clinical symptoms of gastroesophageal reflux [4–6]. Treatment of esophageal problems in PD still remains difficult. However, symptoms derived from gastroesophageal reflux can be treated with appropriate antireflux measures.

In this study, we investigated the frequency and clinical features of GERD in PD.

## 2. Methods

### 2.1. Subjects and Informed Consent

Patients were consecutively recruited from the outpatient clinic of neurology at the Research Institute for Brain and Blood Vessels, from October 2010 to September 2011. The patients had to fulfill the criteria of the United Kingdom PD Brain Bank [7]. Healthy controls were also consecutively recruited. All the patients and controls were interviewed and neurologically examined and then confirmed as having no systemic or neurological disorder. Written informed consent to participate in this study was obtained from all the participants.

### 2.2. Institutional Approvals and Study Protocols

The Ethical Committee of the Research Institute for Brain and Blood Vessels approved this study. All the participants were questioned about subjective complaints of heartburn. Clinical features of GERD were assessed by completion of a questionnaire, the frequency scale for symptoms of GERD (FSSG); the details are described elsewhere [8, 9]. In brief, FSSG is the Japanese GERD questionnaire scored to indicate the frequency of symptoms (0 = never, 1 = occasionally, 2 = sometimes, 3 = often, and 4 = always) that can be used to diagnose GERD when the total FSSG score is more than 8. FSSG was translated into English by the original authors (Table 1) [8]. We enrolled the participants who scored 22 or more on the Mini-Mental State Examination (MMSE) and provided adequate responses to our questions during interviews. Clinical severities of parkinsonism were evaluated by disease duration, Hoehn and Yahr (H&Y) stage, and unified PD rating scale (UPDRS). Forward-bent abnormal postures of the body trunk were considered as bent forward flexion which can induce GERD if the abnormal posture caused deterioration of any part of the patients' daily lives. MMSEs were completed by conventional face-to-face neuropsychological assessments. The PD questionnaire-39 (PDQ-39) was answered by the patients themselves, and the nonmotor symptoms score (NMSS) was completed by the patients according to the physician's instructions. The other demographical parameters were obtained from all the participants. 

### 2.3. Statistical Analysis

We first investigated prevalence rates of subjective heartburn and determined FSSG scores in the patient-control study. We compared the frequency of subjective heartburn using the chi-square test and FSSG scores using the Mann-Whitney *U* test. In the analysis of FSSG, we compared the total score and each item using the Mann-Whitney *U* test. Following this, we compared clinical characteristics of PD with or without GERD and examined the correlation of these characteristics with FSSG scores using Spearman's rank correlation coefficient. Finally, we performed multiple logistic regression analysis of PD with or without GERD. In the analysis, we used “1” to represent PD with GERD, males, and patients with the wearing-off phenomenon and “0” to represent PD without GERD, females, and patients without the wearing-off phenomenon. The other parameters were represented by their original values.

## 3. Results

### 3.1. Patient-Control Study of GERD

**Figure 1:**
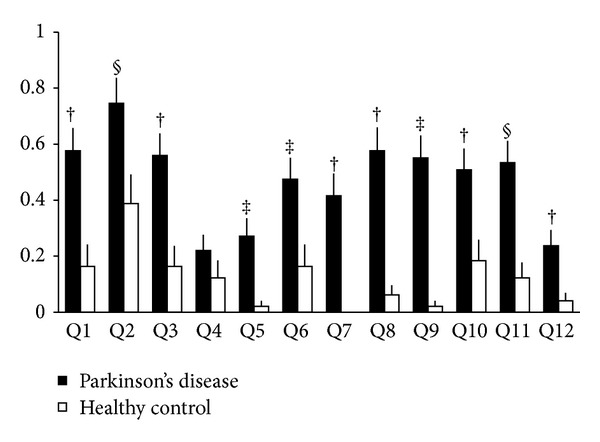
Results for each item of the frequency scale for symptoms of gastroesophageal reflux disease. All values are shown as means and standard errors. Each symbol indicates the *P* value: ^§^ *P* < 0.05, ^‡^ *P* < 0.01, and ^†^ *P* < 0.001.

In this study, we recruited 169 participants (120 PD patients and 49 healthy controls) for 12 months of registration. Healthy controls had no routine medicine and critical disease at all. PD patients also had no medical history on gastrointestinal diseases, whereas 3 PD patients had antacid medication. Eighteen PD patients had MMSE scores less than 22. Their demographic characteristics are shown in Table 2. There were no statistical differences in the gender rate and age between the 2 groups. Bent forward flexion was observed in 23 (22.5%) PD patients and 1 (0.02%) healthy control (*P* < 0.01). Subjective heartburn complaints were expressed by 22 (20.6%) PD patients and 4 (8.2%) healthy controls. The prevalence rate in the PD patients was significantly higher than that in the healthy controls (*P* = 0.04). The FSSG score (mean ± SD) was 5.9 ± 7.2 for the PD patients and 1.4 ± 3.1 for the healthy controls. The FSSG score of the PD patients was significantly higher than that of the healthy controls (*P* < 0.0001). In total, 27 (26.5%) PD patients and 4 (8.2%) healthy controls scored more than 8 on FSSG. Each item of the FSSG was also significantly higher in PD patients than in healthy controls except for question 4 as shown in Figure 1, in which subjects were asked about subconsciously rubbing their chest with their own hands. The odds ratio of GERD defined by an FSSG score of more than 8 in the PD patients compared with that in the healthy controls was 4.05 (95% confidence interval: 1.33–12.33, *P* < 0.01); however, there were no statistically significant differences in the gender and age within our study cohort.

### 3.2. Statistical Analysis of Differences between PD with and without GERD

In PD, a patient with GERD and a patient without GERD had H2 blocker, 20 mg per day of famotidine, and a patient with GERD had proton pump inhibitor, 30 mg per day of lansoprazole. The demographic characteristics of PD patients with GERD are shown in Table 3. There were no statistical differences in the gender rate, age, onset age of PD, dopaminergic treatment calculated as the levodopa equivalent daily dose, and MMSE between the PD patients with or without GERD. Subjective heartburn was observed in 44.4% PD patients with GERD and was significantly frequent (*P* < 0.001). Bent forward flexion was frequent in the PD patients with GERD (*P* = 0.04). There were no statistical differences in the disease duration, H&Y stage, and UPDRS part III; however, the UPDRS total and part II scores were significantly higher (*P* = 0.02 and *P* < 0.001, resp.), the wearing-off phenomenon was significantly frequent (*P* < 0.01), PDQ-39 was significantly higher (*P* < 0.001), and NMSS was significantly higher in the PD patients with GERD (*P* < 0.0001).

### 3.3. Correlation between the FSSG Score and Clinical Characteristics of PD

Positive correlations were determined between the FSSG score and the disease duration (*P* = 0.03), UPDRS part II (*P* < 0.01), PDQ-39 (*P* < 0.001), and NMSS (*P* < 0.0001). Spearman's rank correlation coefficients were 0.22, 0.31, 0.36, and 0.46, respectively. There were no significant relationships between the FSSG score and age, H&Y stage, total UPDRS, and UPDRS part III.

### 3.4. Independent Factors Related to the Presence of GERD in PD

The results of multiple logistic regression analysis of GERD adjusted by the motor score, presented in Table 4, showed that the significant independent factors related to the presence of GERD in PD were UPDRS part II (odds ratio = 1.35, 95% confidence interval: 1.03–1.77, *P* = 0.03) and NMSS (odds ratio = 1.05, 95% confidence interval: 1.00–1.10, *P* = 0.05). Other factors, including the gender, age at this study, disease duration, wearing-off phenomenon, and PDQ-39 score, showed no statistical significance. These results did not change after adjustments for gender or age.

## 4. Discussion

Our patient-control study suggested that GERD, as defined by the FSSG score, was more prevalent in PD patients than in the healthy controls. The prevalence rate was 26.5%. The presence of PD increased the prevalence rate of GERD to 4.1 times higher than that of the age-matched controls. These findings indicated that PD can be a risk factor of GERD. The results of the analysis comparing between PD with and without GERD suggested that GERD was clinically characterized by subjective heartburn and was more common in the advanced stage presenting with the wearing-off phenomenon. The analysis also suggested that GERD could cause deterioration of patients' daily living activities and quality of life and that GERD was associated with the presence of other nonmotor symptoms. Furthermore, daily living activity and nonmotor symptoms can be independent relating factors of GERD in PD. These results suggest that GERD is a frequent nonmotor problem and a deteriorating factor of daily living activity in PD patients. Because clinical symptoms of GERD are treatable, efforts to recognize the presence of GERD should be made to preserve the quality of life of PD patients. However, there remains a certain limitation to interpret the results. We cannot exclude the possibility that our results were peculiar to the outpatient.

Although PD is still the most well-known movement disorder, growing recognition of variable nonmotor symptoms suggests that PD is a systemic disease. Nonmotor symptoms of PD are a major cause of disability for PD patients, and recognition and treatment of nonmotor symptoms are important to maintain comprehensive healthcare for PD patients [3, 10, 11]. Variable symptoms in the alimentary system from the mouth to the anorectum have been reported [2]. Gastrointestinal problems are also a type of nonmotor symptoms. All parts of the gastrointestinal tract can be affected, even in the earlier phase of the disease course in some cases. Nonmotor symptoms are troublesome for PD patients and physicians because conventional dopaminergic therapy does not always work efficiently in the management of these symptoms. Therefore, physicians should be alert for treatable symptoms.

The etiology of GERD still remains unclear; however, pathological research has provided some suggestive findings regarding the alimentary system. Lewy bodies in the extra central nervous system have been reported in Auerbach's and Meissner's plexuses by systemic pathological examination [12]. The lower esophagus is one of the extra central nervous organs that share Lewy bodies, which are frequently found in Auerbach's plexus. Pathological abnormalities may induce variable degrees of functional disorders in the lower esophagus. Although Lewy bodies in the alimentary system have been reported in autopsy cases with megacolon and achalasia [13–15], there is no direct evidence of the association between GERD and the lower esophageal Lewy bodies. However, these previous reports reasonably support that pathological abnormality of the lower esophagus may cause the clinical symptoms of GERD in PD. 

There are research data indicating the relationship between spinal kyphosis associated with osteoporosis and GERD in elderly people [16–18]. Postural abnormalities of the trunk are also frequent in PD patients [19]. The bent forward postural abnormality, known as camptocormia, is one such trunk abnormality that occurs in PD patients [20]. The prevalence rate varies from 3% to 17.6% [19, 21–24]. We defined patients who had this postural abnormality, regardless of the degree of thoracolumbar flexion, and also complained of distress in their daily life because of the abnormal posture as having bent forward flexion because there are no unified and confirmed diagnostic criteria for abnormal posture in PD. In our study, the prevalence rate of bent forward flexion using this definition was relatively frequent compared with that in previous reports. 

Dopaminergic agents induce gastrointestinal problems by stimulating the peripheral dopaminergic receptors, which are mainly induced as nausea. A clinical review that has described adverse effects of dopaminergic agonists has addressed nausea as a popular adverse effect in the early stage of PD patients, whereas GERD or gastroesophageal influx has not been mentioned [25]. Although nausea is a common clinical symptom of GERD, there is no evidence that nausea leads to GERD. As shown in the present results, there were no differences in the use of dopaminergic agents expressed as the levodopa equivalent daily dose between PD with GERD and without GERD, which indicates that dopaminergic agents are not directly linked to the development of GERD. 

*Helicobacter pylori* (*H. pylori*) infection can induce motor fluctuations by interrupting the absorption of levodopa in PD patients [26–29]. Eradication of *H. pylori* can improve these annoying problems in *H. pylori*-infected PD patients. However, it is unpredictable whether *H. pylori* eradication is helpful for improving symptoms of GERD in PD patients because the effect of eradication is still controversial in patients with GERD [30–32].

The diagnosis of GERD is commonly based on the history or findings from upper gastrointestinal endoscopy. As a therapeutic diagnostic method, 24 h esophageal pH monitoring combined with the PPI test [33] is also used. Because clinical history-based diagnosis is the simplest and quickest, demanding no additional workload of the patients, it is suitable for clinical practice. FSSG was created in Japan for physicians, including general practitioners, to not only assist in the initial diagnosis of GERD, but also allow quantitative assessment of the effects of treatment and the changes in symptoms over time [8]. A significant reduction in the FSSG score occurs in patients with both mild and severe GERD after therapy with PPI [9]. FSSG contains the 12 symptoms most commonly experienced by GERD patients, with 7 being reflux symptoms and the remaining 5 being dyspeptic symptoms. When the total score is more than 8, GERD can be diagnosed with 62% sensitivity and 59% specificity.

## Figures and Tables

**Table 1:** The frequency scale for symptoms of gastroesophageal reflux disease.

	Frequency				
	Never	Occasionally	Sometimes	Often	Always
(1) Do you get heartburn?	0	1	2	3	4
(2)Does your stomach get bloated?	0	1	2	3	4
(3)Does your stomach ever feel heavy after meals?	0	1	2	3	4
(4)Do you sometimes subconsciously rub your chest with your hand?	0	1	2	3	4
(5)Do you ever feel sick after meals?	0	1	2	3	4
(6)Do you get heartburn after meals?	0	1	2	3	4
(7)Do you have an unusual (e.g., burning) sensation in your throat?	0	1	2	3	4
(8)Do you feel full while eating meals?	0	1	2	3	4
(9)Do some things get stuck when you swallow?	0	1	2	3	4
(10) Do you get bitter liquid (acid) coming up into your throat?	0	1	2	3	4
(11) Do you burp a lot?	0	1	2	3	4
(12) Do you get heartburn if you bend over?	0	1	2	3	4

**Table 2:** Demographic characteristics of the participants.

	Parkinson's disease	Healthy controls	*P* value
	(*n* = 102)	(*n* = 49)	
Gender, *n* (%)			
Female	58 (56.9)	25 (51.0)	NS
Male	44 (43.1)	24 (49.0)	
Age, y, mean ± SD (range)	70.3 ± 8.3 (48–91)	70.7 ± 6.7 (59–85)	NS
Bent forward flexion, *n* (%)	23 (22.5)	1 (0.02)	<0.01
Heartburn, *n* (%)	21 (20.6)	4 (8.2)	0.04
FSSG			
Mean ± SD (range)	5.9 ± 7.2 (0–37)	1.4 ± 3.1 (0–14)	<0.0001
More than 8, *n* (%)	27 (26.5%)	4 (8.2%)	<0.01

NS: no significance; FSSG: the frequency scale for symptoms of GERD (GERD: gastroesophageal reflux disease).

**Table 3:** Demographic characteristics of Parkinson's disease with or without GERD.

	Parkinson's disease with GERD (*n* = 27)	Parkinson's disease without GERD (*n* = 75)	*P* value
Gender, *n* (%)			
Female	16 (59.3)	42 (56.0)	NS
Male	11 (40.7)	33 (44.0)	NS
Age, y, mean ± SD (range)	69.8 ± 9.5 (56–91)	70.4 ± 7.9 (48–88)	NS
Onset age, y, mean ± SD (range)	62.6 ± 9.8 (38–78)	64.5 ± 9.2 (40–83)	NS
LEDD, mg, mean ± SD	766.3 ± 449.9	653.7 ± 334.0	NS
MMSE, mean ± SD	26.9 ± 2.8	26.4 ± 2.4	NS
Heartburn, *n* (%)	12 (44.4)	10 (13.3)	<0.001
Bent forward flexion, *n* (%)	10 (37.0)	13 (17.3)	0.04
Disease duration, m, (range)	91.6 ± 50.5 (12–241)	76.2 ± 49.5 (6–218)	NS
H&Y stage, mean ± SD	2.2 ± 0.8	2.2 ± 0.9	NS
Stage 0, *n* (%)	0 (0)	2 (2.7)	
Stage 1, *n* (%)	5 (18.5)	10 (13.3)	
Stage 2, *n* (%)	13 (48.1)	40 (53.3)	
Stage 3, *n* (%)	8 (29.6)	17 (22.7)	
Stage 4, *n* (%)	1 (3.7)	6 (8.0)	
UPDRS			
Total, mean ± SD (range)	35.3 ± 13.9 (1–56)	28.4 ± 14.3 (3–73)	0.02
Part II, mean ± SD (range)	14.6 ± 7.0 (5–29)	8.8 ± 6.2 (0–25)	<0.001
Part III, mean ± SD (range)	17.0 ± 8.7 (4–36)	17.6 ± 10.1 (0–46)	NS
Wearing-off phenomenon, *n* (%)	13 (48.1)	16 (21.3)	<0.01
PDQ-39, mean ± SD (range)	56.3 ± 31.7 (3–134)	29.8 ± 24.7 (0–108)	<0.001
NMSS, mean ± SD (range)	50.9 ± 34.0 (9–135)	22.9 ± 22.4 (0–117)	<0.0001

NS: no significance; GERD: gastroesophageal reflux disease; LEDD: levodopa equivalent daily dose; MMSE: Mini-mental Examination Test; H&Y: Hoehn and Yahr; UPDRS: unified Parkinson's disease rating scale; PDQ-39: Parkinson's disease questionnaire-39; NMSS: nonmotor symptom scale.

**Table 4:** Results of multiple logistic regression analysis of GERD in Parkinson's disease.

	Odds ratio	95% CI	*P* value
Gender	0.68	0.09–5.32	NS
Age	1.95	0.31–12.09	NS
Disease duration	1.00	0.99–1.01	NS
Wearing-off phenomenon	3.47	0.37–32.85	NS
UPDRS part II	1.35	1.03–1.77	0.03
PDQ-39	1.00	0.94–1.05	NS
NMSS	1.05	1.00–1.10	0.05

NS: no significance; GERD: gastroesophageal reflux disease; CI: confidence interval; UPDRS: unified Parkinson's disease rating scale; PDQ-39: Parkinson's disease questionnaire-39; NMSS: nonmotor symptom scale.
